# Effect of beraprost sodium on arterial stiffness in patients with type 2 diabetic nephropathy

**DOI:** 10.1186/1745-6215-14-275

**Published:** 2013-09-02

**Authors:** Ki Young Na, Dong Ki Kim, Sung Gyun Kim, Young-Ki Lee, Chun Soo Lim

**Affiliations:** 1 Seoul National University Bundang Hospital, 82 Gumi-ro 172 Beon-gil, Seongnam-si, Gyeonggi-do 463-707, South Korea; 2 Department Internal Medicine, Seoul National University College of Medicine, 103 Daehak-ro, Jongno-gu, Seoul 110-799, South Korea; 3 Seoul National University Hospital, 101 Daehak-ro Jongno-gu, Seoul 110-744, South Korea; 4 Hallym University Sacred Heart Hospital, 896 Pyeongchon-dong, Dongan-gu, Anyang, Gyeonggi-do 431-070, South Korea; 5 Kangnam Sacred Heart Hospital, 948-1 Daerim1 dong, Yeongdeungpo-gu, Seoul 150-950, South Korea; 6 Seoul National University Boramae Medical Center, 20 Boramae-ro 5-gil, Dongjak-gu, Seoul 156-707, South Korea

**Keywords:** Beraprost sodium, Arterial stiffness, Diabetic nephropathy, Cardiovascular, Pulse wave velocity

## Abstract

**Background:**

Diabetic nephropathy is the leading cause of end-stage renal disease (ESRD). Cardiovascular (CV) complications are the most common cause of death among ESRD patients. Beraprost sodium (BPS) is a prostacyclin analog with vasodilatory and antiplatelet effects.

**Methods:**

This is a multicenter prospective, randomized, double-blind, placebo-controlled trial to determine whether treatment with BPS improves arterial stiffness in patients with type 2 diabetic nephropathy. A total of 102 participants with type 2 diabetic nephropathy will be screened, enrolled, and randomly assigned to receive either 80 μg BPS or placebo daily for 12 weeks. The primary outcome is the change in brachial-ankle pulse wave velocity between baseline and after 12 weeks of medication use. The secondary outcomes will include changes in the ankle-brachial index, the urine albumin to creatinine ratio, the estimated glomerular filtration rate, lipid profiles, and blood pressure from baseline to after treatment.

**Discussion:**

This clinical trial is the first to investigate the effects of BPS on changes in CV biomarkers, albuminuria, renal function, and lipid profiles in patients with diabetic nephropathy.

**Trial registration:**

ClinicalTrials.gov number NCT01796418

## Background

The impact of diabetic nephropathy on the increasing population with chronic kidney disease (CKD) and end-stage renal disease (ESRD) is enormous. Diabetic nephropathy has become the leading cause of ESRD and is responsible for more than 40% of all cases in the USA and South Korea [1,2]. Although randomized studies have demonstrated that intensified multifactorial intervention in patients with type 2 diabetes reduces the risk of microangiopathy, cardiovascular (CV) events, and mortality [3-5], the incidence of ESRD due to diabetes is relentlessly increasing worldwide. CV complications are the number one cause of death among patients with ESRD [1,2]. Moreover, CV risk is not limited to ESRD, and the risk of CV mortality begins to increase with even a mild impairment of kidney function [6]. Therefore, early detection and intervention for CV complications are important to reduce mortality among patients with CKD or ESRD due to diabetes. In this regard, surrogate markers precisely estimating CV risk are essential. Pulse wave velocity (PWV) and the ankle-brachial index (ABI) are useful noninvasive biomarkers for predicting CV risk [7]. PWV provides a measure of large artery stiffness and has been reported as a risk factor for nonfatal CV disease and CV mortality independent of systolic blood pressure in dialysis patients [8,9]. The ABI is a clinical tool for the detection of peripheral arterial disease and the prediction of CV mortality and morbidity [10].

Beraprost sodium (BPS) is a stable orally active prostacyclin (PGI_2_) analog. BPS acts by binding to PGI_2_ membrane receptors and inhibiting the release of Ca^2+^ from intracellular storage sites. These effects lead to the relaxation of the smooth muscle cells and vasodilation. BPS has also been suggested to improve microvascular circulation through the reduction of red blood cell deformability. Moreover, BPS alleviates vascular thrombosis by inhibiting platelet aggregation. Through these vasodilatory and antiplatelet effects, BPS appears to be an effective agent in the treatment of patients with pulmonary arterial hypertension, Buerger’s disease, and arteriosclerosis obliterans [11].

An *in vitro* experiment showed that BPS improved endothelial function through the induction of nitric oxide (NO) production and endothelial NO synthase activation in endothelial cells [12]. In addition, BPS lowered serum vascular cell adhesion molecule-1 levels and prevented the increase of carotid intima-media thickness in patients with type 2 diabetes, which suggested a beneficial effect of BPS on the progression of atherosclerosis in diabetic patients [13]. The development of arterial stiffness, as assessed by PWV, has been prevented by BPS treatment in elderly patients with cerebral infarction [14,15]. BPS has also been shown to improve dyslipidemia and metabolic syndrome in diabetic animal models [16,17]. A recent study has reported that 8 weeks BPS treatment improves pain in diabetic patients with painful peripheral neuropathy [18]. However, no studies have observed the effects of BPS on CV biomarkers in diabetic nephropathy patients with a high risk of CKD or ESRD.

Based on reports of the beneficial effects of BPS, we hypothesize that BPS treatment will improve arterial stiffness in diabetic nephropathy patients. To evaluate this hypothesis, we will conduct this clinical trial in patients with type 2 diabetic nephropathy.

## Methods

### Hypothesis

Treatment with BPS will improve arterial stiffness in patients with type 2 diabetic nephropathy. Compared with placebo-treated patients, arterial PWV will decrease in BPS-treated patients.

### Study design

**Figure 1:**
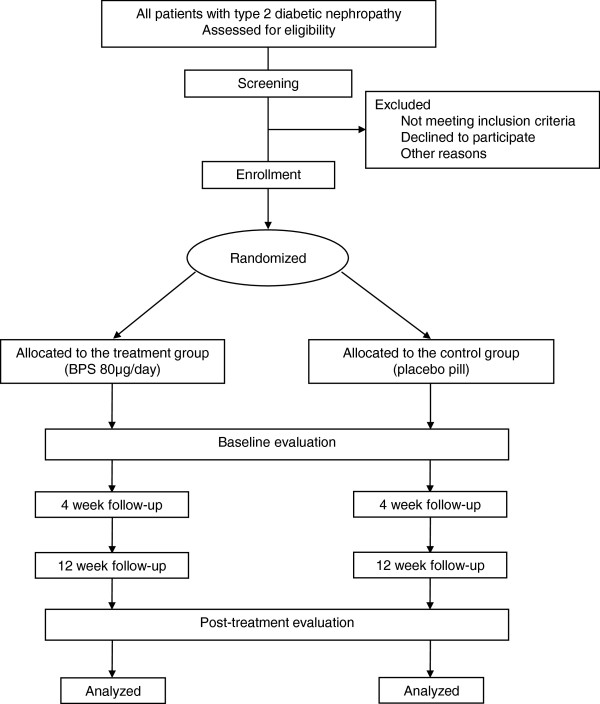
**Study algorithm.** BPS, beraprost sodium.

This is a multicenter prospective, randomized, double-blind, placebo-controlled trial. This study is an investigator-initiated clinical trial. The study algorithm is shown in Figure 1. After enrollment and treatment, clinical follow-up will be conducted after 4 and 12 weeks of treatment.

### Study participants

All patients aged 20 to 75 years with type 2 diabetes mellitus will be screened. The following will be performed at the initial visit: 1) questionnaire regarding past medical history and drug history; 2) physical examination of all systems; 3) measurement of height and weight; 4) blood pressure and pulse rate measurement; and 5) collection of blood and urine. Patients who meet all of the inclusion and exclusion criteria and provide written informed consent are eligible for this study (Table 1).

**Table 1:** Inclusion and exclusion criteria

**Inclusion criteria**	**Exclusion criteria**
Age between 20 and 75 years	History of kidney transplantation
Type 2 diabetes using a glucose-lowering agent or insulin	Advanced congestive heart failure (≥ NYHA class III)
Estimated GFR by the IDMS MDRD equation ≥30 ml/min/1.73 m^2^	Uncontrolled arrhythmia
More than two UACR ≥30 mg/g or UPCR ≥ 300 mg/g in the most recent 6 months	Advanced liver cirrhosis (Child-Pugh class C)
Blood pressure ≤140/90 mmHg without any additional antihypertensive medications in the most recent 3 months	History of bleeding diathesis
	Active infection or uncontrolled inflammatory disorders
	History of cerebrovascular accident or myocardial infarction
	Current use of an anticoagulant
	Current use of more than two antiplatelet agents
	Advanced malignancy (life expectancy ≤6 months)
	Uncontrolled diabetes (HbA1C >10%)
	Severe anemia (hemoglobin <8.0 g/dL)
	Pregnancy or lactation
	Genetic disease including galactose intolerance, lactose deficiency, and glucose-galactose malabsorption

GFR, glomerular filtration rate; HbA1C, hemoglobin A1C; IDMS, isotope dilution mass spectrometry; MDRD, Modification of Diet in Renal Disease; NYHA, New York Heart Association; UACR, urine albumin to creatinine ratio; UPCR, urine protein to creatinine ratio.

Serum and urine creatinine (Cr) will be measured using the isotope dilution mass spectrometry-traceable method on a Toshiba TBA 200FR Analyzer (Toshiba, Tokyo, Japan). Urinary albumin will be measured using an immunoturbidimetric assay on a Toshiba TBA 120FR Analyzer. The estimated glomerular filtration rate (eGFR) is calculated using the Modification of Diet in Renal Disease formula. The formula was defined as follows, where the serum Cr concentration is in mg/dL: eGFR = 175 × Cr^−1.154^ × Age^−0.203^ × (0.742 if female) [19].

### Randomization

A research coordinator will perform the randomization and deliver the study drugs. The participants and investigators will be blinded to the treatment assignment. A list of random numbers will be generated by an independent statistician and then used to randomly allocate the treatments to equally sized treatment and control groups. The randomization will be stratified on the basis of sex and age, which will utilize a randomized block design in blocks of four patients in the order of enrollment in each arm. A randomization code list will be generated by a third party - the medical statistics support team in the Seoul National University Boramae Medical Center. The treatments will be allocated to the participants in accordance with the predefined randomization list (1:1 ratio for the treatment and control groups).

### Treatments

After randomization, the participants will take either a BPS (treatment group) or placebo (control group) pill during the study. The tablet shapes and packaging of the placebo pills are indistinguishable from those of the original BPS pills. The BPS and placebo tablets are provided by Astellas Pharma Korea, Inc. (Seoul, Korea). The prescription and administration of the study drugs will be performed in a double-blind manner. Based on the report showing the prevention of arterial stiffness with BPS [14], 80 μg/day BPS in two divided doses will be administered to the participants for 12 weeks. The administration of other prostacyclin agents, such as limaprost alfadex, or fluorinated pyrimidine antifungal agents, such as flucytosine, will be prohibited during the study. Participants with any changes in antihypertensive, antiplatelet, or anticoagulant medications during the study will be dropped. Investigators or research coordinators will evaluate drug compliance in the study participants, and participants with less than 80% compliance will be dropped from the study.

### Outcome measures

The primary outcome to be measured is the change in the brachial-ankle PWV (ba-PWV) between baseline and after 12 weeks of treatment. The secondary outcomes will include changes in the ABI, urine albumin to creatinine ratio, eGFR, lipid profiles, and blood pressure from baseline to after treatment.

### Clinical and laboratory evaluations

**Figure 2:**
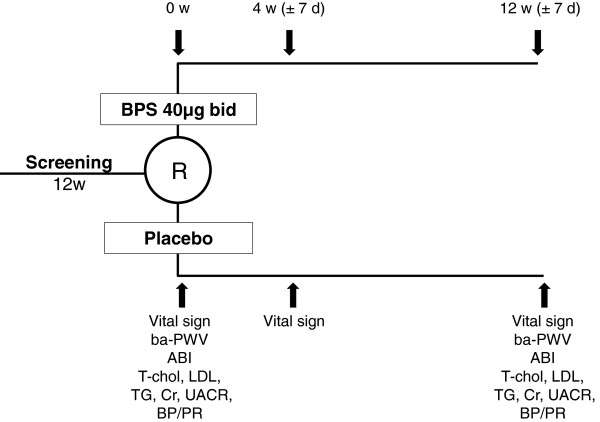
**Study schedule.** ABI, ankle-brachial index; ba-PWV, brachial-ankle pulse wave velocity; BP, blood pressure; BPS, beraprost sodium; Cr, creatinine; d, days; LDL, low-density lipoprotein; PR, pulse rate; R, randomization; T-chol, total cholesterol; TG, triglycerides; UACR, urine albumin to creatinine ratio; w, weeks.

The physical examination and medication reviews will be performed before treatment and after 4 and 12 weeks of treatment. The laboratory evaluations, including a complete blood count, Cr, total cholesterol, triglyceride, low-density lipoprotein cholesterol, random urine albumin, and urine Cr, will be performed at baseline and after 12 weeks of treatment. ba-PWV and ABI will be measured using a Colin pulse waveform analyzer (Colin CO, Ltd, Komaki, Japan) at baseline and after 12 weeks of treatment. The study schedule is shown in Figure 2.

### Monitoring and safety assessments

Before the start of this trial, a central monitor will review the study protocol and clinical research form (CRF) with the investigators and research coordinators. During the study period, a monitor will regularly visit each institute and evaluate the records of the participants, the CRF, compliance with the study protocol and Good Clinical Practice guidelines, and enrollment status. The storage, delivery, and accountability of the study drug will also be monitored during this period.

Any presence of bleeding, headache, hot flushes, shock, interstitial pneumonia, abnormal liver function, hypersensitivity reaction, angina, or gastrointestinal discomfort will be recorded during treatment with BPS/placebo. All adverse events, including serious adverse events (SAEs), will be recorded and followed-up for the duration of the study or until resolution. The assessment of adverse events will be performed by investigators and research coordinators. All SAEs will be graded and reported to the principal investigator and the ethics committee.

### Sample size calculations

No previous report has evaluated the effect of BPS on arterial stiffness in diabetic patients. We therefore referred to a study showing that the mean and standard deviation of the PWV change due to BPS treatment in patients with a history of cerebrovascular accidents were 260 cm/s and 345 cm/s, respectively [14]. We adjusted the sample size for an estimated drop-out rate of 25% due to poor compliance, a two-sided level of significance of α = 5%, and a power of 1-β = 90%, which indicated that 51 participants will be required in each group to detect a significant difference with a two-sided Student’s *t* test [20]. A total of 102 participants will be randomized and included in the analysis.

### Statistical analyses

The statistical analyses will be performed both on per protocol (PP) and intention-to-treat (ITT) bases. For the PP analysis, all participants who completed the study and were evaluated for the primary outcome will be included. For the ITT analysis, all participants who signed the written informed consent and were randomized into the study will be included, regardless of whether the correct treatment was administered. To evaluate the primary outcome, a PP analysis will be performed for those participants who showed a primary outcome variable among the targets for the PP analysis. The secondary outcomes will be evaluated in the participants who completed the study. For the safety evaluation, all participants who signed the written informed consent and were randomized to receive at least one of the active treatments (BPS/placebo medication) will be included in the analysis. The safety analysis will include the calculation of the frequencies and rates of complications and SAEs reported in the two groups.

The baseline clinical characteristics and laboratory data of the study participants will be summarized in terms of frequencies and percentages for categorical variables and means with standard deviations for continuous variables. Continuous variables will be analyzed using Student’s *t* test or the Mann–Whitney U test. Chi-squared test or Fisher’s exact test will be used for categorical variables. Differences in the changes in the outcome measurement variables between the treatment and control groups will be tested with one-way analysis of variance. Differences in the temporal changes in the outcome measurement variables in each group will be analyzed with the paired *t* test or Wilcoxon signed rank test. A value of *P* < 0.05 will be considered to be statistically significant. All analyses will be performed using SPSS (version 16.0. for Windows; SPSS Inc., Chicago, IL, USA).

### Ethical approval

This study will be performed in accordance with the Declaration of Helsinki amended by the 59th World Medical Association General Assembly in 2008. All of the participants will sign a written informed consent stating that participation is voluntary and that participation can be withdrawn at any time, without any negative consequences concerning their current or future medical treatment. This study was approved by the Institutional Review Board of Seoul National University Bundang Hospital, Seoul National University Hospital, Seoul National University Boramae Medical Center, Hallym University Sacred Heart Hospital, and Kangnam Sacred Heart Hospital. The trial protocol has been registered at http://www.clinicaltrials.gov (NCT01796418).

## Discussion

Diabetic nephropathy is the number one cause of ESRD. CV complications are the most common cause of death in ESRD. Therefore, the early detection of CV risk using biomarkers and the prevention of CV risk with an effective drug are the only ways to improve the clinical outcomes and mortality in patients with diabetic nephropathy. BPS, a PGI_2_ analog, has shown many beneficial effects in both preclinical and clinical studies. In this trial, we will test whether BPS improves CV risk. This trial is the first to investigate the effects of BPS on changes in CV biomarkers, albuminuria, renal function, and lipid profiles in patients with diabetic nephropathy.

Because BPS has the potential to increase bleeding risk by inhibiting platelet aggregation, even patients on a single antiplatelet agent appear to be in danger of bleeding after their enrollment in this study. However, higher doses of BPS administered for a longer duration compared with this study were previously reported to not increase bleeding risk. According to the study performed by Mohler and colleagues [21], daily intake of 120 μg BPS with other antiplatelet agents (aspirin, clopidogrel, or ticlopidine) for 1 year was not associated with any bleeding event. Furthermore, we will monitor and follow-up all adverse events, including bleeding, for the duration of the study or until resolution.

A 12-week study may be too short to demonstrate a beneficial effect of BPS. To assess the sustained effects of BPS on vascular benefits, long-term studies may be needed. However, in New York Heart Association functional class II and III patients with pulmonary arterial hypertension, a 12-week treatment with BPS improved exercise capacity and symptoms [22]. Moreover, the main mechanism of BPS in the improvement of arterial stiffness is through NO production, and BPS has a short half-life [12]. We therefore hypothesize that short-term treatment with BPS will improve arterial stiffness in this study. If the beneficial effects of BPS are proven, BPS could be used in clinical practice to improve CV and renal outcomes in this high-risk population. Future clinical trials with an extended administration of BPS over 52 weeks in a larger population will be required to determine whether BPS improves the actual CV morbidity and mortality.

## Abbreviations

ABI: Ankle-brachial index; ba-PWV: Brachial-ankle pulse wave velocity; BPS: Beraprost sodium; CKD: Chronic kidney disease; Cr: Creatinine; CRF: Clinical research form; CV: Cardiovascular; eGFR: estimated glomerular filtration rate; ESRD: End-stage renal disease; ITT: Intention-to-treat; NO: Nitric oxide; PGI2: Prostacyclin; PP: Per protocol; PWV: Pulse wave velocity; SAE: Serious adverse events.

## Competing interests

The authors declare that they have no competing interests.

## Authors’ contributions

KYN participated in the design of the study and drafted the manuscript. DKK conceived of the study, participated in the design of the statistical analysis, and helped to draft the manuscript. SGK and YKL participated in the design of the study. CSL conceived of the study, participated in acquiring funding, and had final responsibility for the decision to submit for publication. All authors read and approved submission of the final manuscript.
